# The obesity paradox for survivors of critically ill patients

**DOI:** 10.1186/s13054-022-04074-1

**Published:** 2022-07-03

**Authors:** Dawei Zhou, Chao Wang, Qing Lin, Tong Li

**Affiliations:** Department of Critical Care Medicine, Beijing Tongren Hospital, Capital Medical University, Beijing, China

**Keywords:** Obesity paradox, Critical care, Survivors, Mortality

## Abstract

The obesity paradox has been observed in short-term outcomes from critical illness. However, little is known regarding the impact of obesity on long-term outcomes for survivors of critically ill patients. We aimed to evaluate the influence of obesity on long-term mortality outcomes after discharge alive from ICU. The adult patients who were discharged alive from the last ICU admission were extracted. After exclusion, a total of 7619 adult patients discharged alive from ICU were included, with 4-year mortality of 32%. The median body mass index (BMI) was 27.2 (IQR 24–31.4) kg/m^2^, and 2490 (31.5%) patients were classified as obese or morbidly obese. The morbidly obese patients had the highest ICU and hospital length of stay. However, higher BMI was associated with lower hazard ratio for 4-year mortality. The results showed the obesity paradox may be also suitable for survivors of critically ill patients.

## Background

Survivors of critically ill patients are at risk of experiencing significant physical, cognitive, and mental health issues, which were associated with an increased mortality following discharge from intensive care unit (ICU) [1–4]. However, factors associated with mortality for survivors of critical illness are not well understood, which may be important when counseling patients and families.

The obesity paradox, which is the phenomenon that obesity increases the risk of obesity-related diseases but paradoxically is associated with survival benefits, has been observed in short-term outcomes from critical illness [5–7]. Nonetheless, little is known regarding the impact of obesity on long-term outcomes after discharge from intensive care unit (ICU).

We, therefore, aimed to evaluate the influence of obesity on 4-year mortality outcome for survivors of critically illness.

## Methods

Data were extracted from the online database Medical Information Mart for Intensive Care (MIMIC III) [8]. We included adult patients who were discharged alive from the last ICU admission. The exclusion criteria were: (1) No weight and height data available, (2) body mass index (BMI) < 10 kg/m^2^ or > 70 kg/m^2^, (3) No follow-up survival data available.

We abstracted the height and weight data from the Electronic Medical Records (EMR) when admitted to ICU. The BMI was calculated using the equation: BMI (kg/m^2^) = weight (kg)/height^2^ (m^2^). BMI was examined as both a categorical and continuous variable. According to the international standards [9], obesity was assessed as a categorical variable according to BMI (BMI less than 10.0 or greater than 70.0 kg/m^2^ was excluded): underweight, BMI < 18.5 k kg/m^2^; normal weight, 18.5 ≤ BMI < 25 kg/m^2^; overweight, 25 ≤ BMI < 30 kg/m^2^; obese, 30 ≤ BMI < 40 kg/m^2^; morbidly obese, BMI ≥ 40 kg/m^2^.

The primary endpoint was 4-year mortality which was extracted from the social security database and was considered as a time-to-event variable.

Baseline and clinical characteristics were compared among different BMI categories. Multiple imputation was used to deal with the missing data with the mice package [10]. We used Cox proportional hazards regression model to produce adjusted hazard ratios (HRs) for the association between BMI and 4-year mortality with normal weight as reference. To better understand the effect of BMI on 4-year mortality as a continuous covariate in multivariable Cox model, the splines-based HR curve was expressed with smoothHR package [11]. The PostgreSQL (version 10, www.postgresql.org) was used for data extraction, and R software (version 3.5.1, www.r-project.org) was used to conduct all the statistical analysis.

## Results

After exclusion, 7619 adult patients discharged alive from ICU were included, with 4-year mortality of 32% (Table 1). The median BMI was 27.2 (IQR 24–31.4) kg/m^2^. 2490 (31.5%) patients were classified as obese or morbidly obese. As expected, higher BMI patients had a higher percentage of comorbidities of hypertension and diabetes mellitus. The morbidly obese patients had the highest ICU and hospital length of stay. The underweight patients had the highest 4-year mortality (62%). The higher BMI was associated with lower HR for 4-year mortality.

**Table 1:** Baseline and clinical characteristics of the study patients by BMI categories

Variables	Total	Underweight (BMI < 18.5 kg/m^2^)	Normal weight (18.5 kg/m^2^ ≤ BMI < 25 kg/m^2^)	Overweight (25 kg/m^2^ ≤ BMI < 30 kg/m^2^)	Obese (30 kg/m^2^ ≤ BMI < 40 kg/m^2^)	Morbidly obese (BMI ≥ 40 kg/m^2^)
Number of patients	7916 (100)	206 (2.6)	2368 (29.9)	2852 (36.0)	2065 (26.1)	425 (5.4)
Age, years	65 (54, 76)	69 (55, 78)	69 (54, 79)	66 (55, 76)	63 (54, 73)	58 (50, 66)
Sex: male	5023 (63)	80 (39)	1371 (58)	2014 (71)	1349 (65)	209 (49)
BMI, kg/m^2^	27.2 (24.0, 31.4)	17.4 (16.4, 18.1)	22.9 (21.5, 24.0)	27.3 (26.2, 28.5)	33.0 (31.4, 35.3)	44.1 (41.7, 48.1)
Ethnicity						
White	5397 (68)	129 (63)	1608 (68)	1951 (68)	1405 (68)	304 (72)
Others	2519 (32)	77 (37)	760 (32)	901 (32)	660 (32)	121 (28)
Marital status						
Married	4373 (55)	80 (39)	1245 (53)	1660 (58)	1171 (57)	217 (51)
Single	1511 (19)	59 (29)	466 (20)	513 (18)	364 (18)	109 (26)
Widowed	1009 (13)	34 (17)	366 (15)	320 (11)	250 (12)	39 (9)
Divorced	469 (6)	13 (6)	111 (5)	169 (6)	142 (7)	34 (8)
Others	554 (7)	20 (10)	180 (8)	190 (7)	138 (7)	26 (6)
Insurance type						
Medicare	4007 (51)	126 (61)	1315 (56)	1418 (50)	983 (48)	165 (39)
Private	3171 (40)	64 (31)	796 (34)	1183 (41)	916 (44)	212 (50)
Medicaid	452 (6)	13 (6)	150 (6)	157 (6)	97 (5)	35 (8)
Government	197 (2)	3 (1)	66 (3)	64 (2)	54 (3)	10 (2)
Self-pay	89 (1)	0 (0)	41 (2)	30 (1)	15 (1)	3 (1)
Comorbidities						
Hypertension	4457 (56)	94 (46)	1169 (49)	1622 (57)	1325 (64)	247 (58)
Diabetes mellitus	2077 (26)	33 (16)	445 (19)	663 (23)	754 (37)	182 (43)
Tumor	525 (7)	22 (11)	182 (8)	175 (6)	119 (6)	27 (6)
Respiratory disease	1212 (15)	52 (25)	375 (16)	357 (13)	321 (16)	107 (25)
CHF	2146 (27)	61 (30)	662 (28)	731 (26)	548 (27)	144 (34)
Liver disease	273 (3)	6 (3)	97 (4)	96 (3)	65 (3)	9 (2)
Renal failure	643 (8)	25 (12)	202 (9)	219 (8)	160 (8)	37 (9)
ICU types						
CCU	1341 (17)	24 (12)	397 (17)	511 (18)	338 (16)	71 (17)
MICU	3803 (48)	64 (31)	1071 (45)	1438 (50)	1062 (51)	168 (40)
CSRU	1191 (15)	67 (33)	379 (16)	368 (13)	289 (14)	88 (21)
SICU	906 (11)	31 (15)	272 (11)	312 (11)	235 (11)	56 (13)
TSICU	675 (9)	20 (10)	249 (11)	223 (8)	141 (7)	42 (10)
ICU scoring systems						
SOFA score	4 (2, 6)	4 (2, 5)	4 (2, 6)	4 (2, 6)	4 (2, 6)	4 (2, 6)
SAPS II	31 (24, 40)	36 (28, 43)	33 (25, 41)	31 (24, 39)	30 (23, 39)	30 (22, 40)
ICU LOS, days	3 (2, 5)	3 (2, 5)	3 (2, 5)	3 (2, 5)	3 (2, 5)	4 (2, 7)
Hospital LOS, days	8 (6, 13)	9 (6, 16)	8 (6, 13)	8 (6, 12)	8 (6, 13)	9 (6, 16)
Hospital discharge location						
Home	1980 (25)	46 (22)	539 (23)	768 (27)	535 (26)	92 (22)
Home health care	3003 (38)	52 (25)	852 (36)	1144 (40)	806 (39)	149 (35)
Rehabilitation center	1364 (17)	44 (21)	441 (19)	420 (15)	355 (17)	104 (24)
Hospice	60 (1)	3 (1)	21 (1)	24 (1)	6 (0)	6 (1)
Skilled nurse facility	1077 (14)	48 (23)	383 (16)	348 (12)	251 (12)	47 (11)
Others	432 (5)	13 (6)	132 (6)	148 (5)	112 (5)	27 (6)
Four-year mortality	2571 (32)	127 (62)	920 (39)	821 (29)	582 (28)	121 (28)

Data are median (interquartile range) or No/Total (%)

*BMI* body mass index, *CHF* chronic heart failure, *CKD* chronic kidney disease, *ICU* intensive care unit, *CCU* Coronary Care Unit, *CSRU* Cardiac Surgery Recovery Unit, *TSICU* Trauma Surgical ICU, *MICU* Medical ICU, *SICU* Surgical ICU, *SOFA* sequential organ failure assessment, *SAPS* Simplified acute physiology score, *LOS* length of stay

**Fig. 1:**
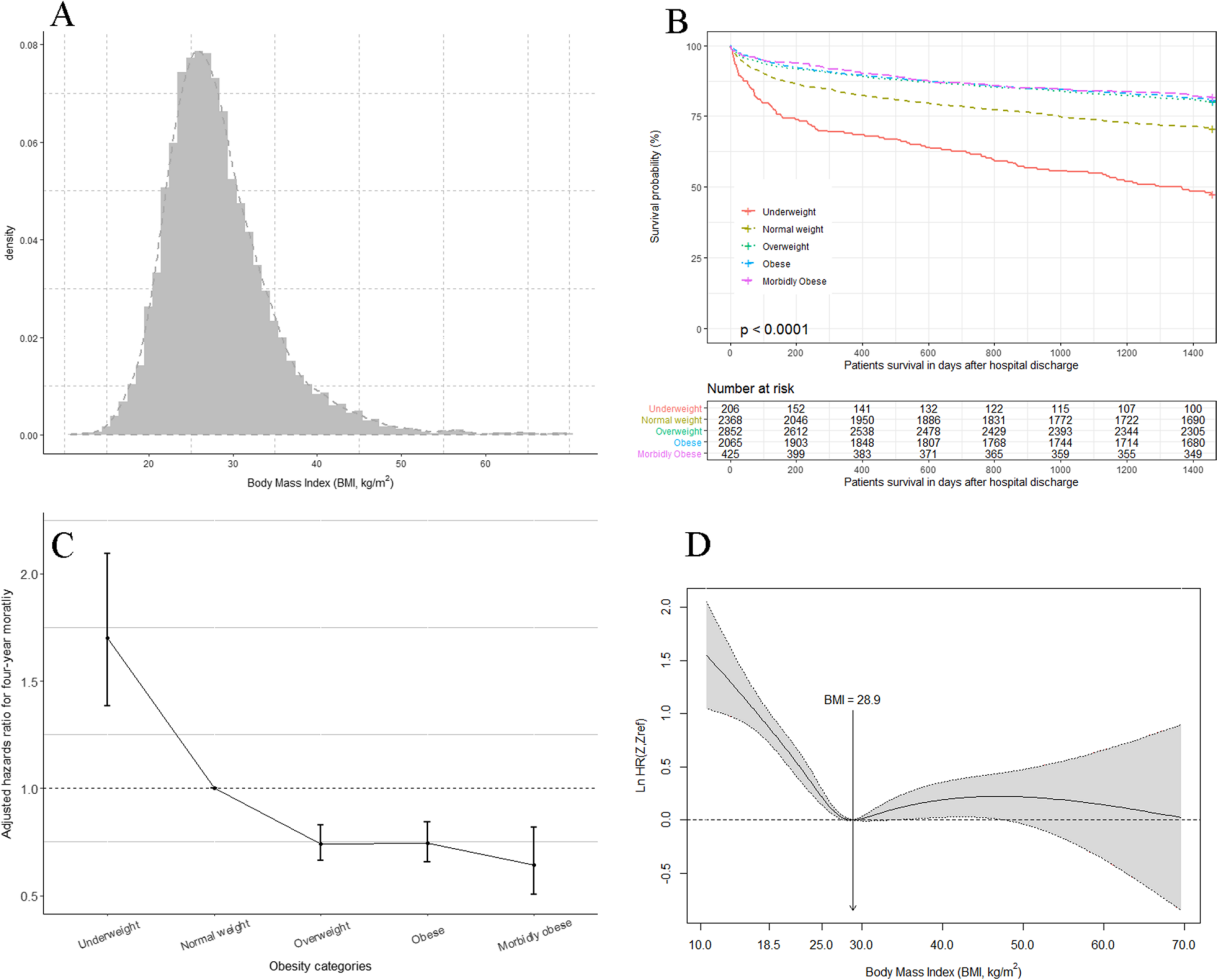
**A** Histogram and density distribution of body mass index (BMI); **B** Kaplan–Meier survival curves by BMI category (log-rank *p* < 0.001); **C** Adjusted hazards ratio (HR) for 4-year mortality according to BMI categories, with normal weight as reference. The HRs and 95% confidence intervals (error bars) for each categories were calculated using COX proportional hazard model after adjusting for age, sex, ethnicity, marital status, insurance type, comorbidities (hypertension, diabetes mellitus, tumor, respiratory disease, chronic heart failure, liver disease, and renal failure), ICU type, disease severity score of simplified acute physiology score (SAPS) II, and hospital discharge locations; **D** Smoothed HR (log transformation) curve with pointwise nonparametric estimation of association between BMI and 4-year mortality after adjusting for confounders (same with the confounders in **C**)

Underweight and normal-weight patients had a lower probability of 4-year survival (Fig. 1B). When considering normal-weight patients as the reference, underweight patients had higher adjusted HR for mortality, while overweight and obese patients had lower adjusted HRs (Fig. 1C). When using BMI as a continuous variable, the pointwise estimation of the adjusted HR curve showed a nonlinear relationship between BMI and long-term mortality outcome, with a BMI of 28.9 kg/m^2^ the lowest HR (Fig. 1D).

## Discussion

In the present study, a significant proportion of patients discharging from ICU survive less than 4 years. Among survivors of critical illness, obesity was associated with lower 4-year mortality.

The high mortality of survivors discharging from ICU was in line with previous studies [12, 13]. In the study of Brinkman et al. [12], the mortality risk at 3 years after hospital discharge was 27.5%; and the 5 years mortality was 26.9% in the study of Doherty et.al, which was higher than the general age-matched population [13]. In view of the prevalence of obesity continuing to rise among the ICU population [6], efforts to understand the impact of obesity long-term outcomes after critical illness should also be important [14]. The present study showed the mortality was high for ICU survivors and obesity was associated with reduced mortality; However, the underlying mechanisms were unknown. Several pathophysiologic mechanisms including higher energy reserves, anti-inflammatory immune profile, role of adipose tissue, and prevention of muscle wasting may contribute to explain this phenomenon [5]. Interestingly, according to the recent study of Drago et al. [15], the relationship of increasing BMI and lower risk of hypoglycemia might contribute to decreased mortality.

The concept of obesity paradox has also been challenged. Martino et.al found that extreme obesity was not associated with a worse survival advantage after adjusted for confounders as well as had a longer duration of mechanical ventilation and ICU length of stay [16]. On the other hand, the BMI as a composite variable is intrinsically problematic, which is not an appropriate measure of fat and skeletal muscle mass and its distribution [17]. In the studies of Jaitovich et al. [18, 19], ICU admission pectoralis muscle area or erector spinae muscle mass was associated with survival outcomes, not was the subcutaneous adipose tissue mass. Similar studies were sparse for ICU survivors. As for survivors of critically ill patients, it is unclear whether it reflects a protective effect or limitations inherent to observational research for obesity paradox, which warrants further research.

There were several limitations to our study. First, the retrospective design in nature was subjected to the inherent limitations even though adjusted for potential confounders. The other confounding factors including smoking status, alcohol consumption, income, education, physical activity, and dietary pattern may be involved, which were not extracted from the database. Second, the ICU admission height and weight data were extracted from the EMR with a higher missing rate. The method (estimation or measurement) for recording the height and weight was unknown. Third, the weight was a variable parameter relying on fluid balance, which could cause perturbation for calculation of BMI. As a result of these limitations, the present findings warrant a cautious interpretation, and more prospective cohort studies are needed to further elucidate this phenomenon.

## Conclusions

In conclusion, our results suggest that the obesity paradox may be also suitable for survivors of critically ill patients.

## Data Availability

Data analyzed during the present study are currently stored in the Medical Information Mart for Intensive Care (MIMIC III) (mimic.mit.edu).

## Abbreviations

BMI: Body mass index
EMR: Electronic medical records
HR: Hazard ratio
ICU: Intensive care unit
MIMIC: Medical Information Mart for Intensive Care
